# Inhibiting miR-205 Alleviates Cardiac Ischemia/Reperfusion Injury by Regulating Oxidative Stress, Mitochondrial Function, and Apoptosis

**DOI:** 10.1155/2021/9986506

**Published:** 2021-06-29

**Authors:** Yuerong Xu, Wangang Guo, Di Zeng, Yexian Fang, Runze Wang, Dong Guo, Bingchao Qi, Yugang Xue, Feng Xue, Zuolin Jin, Yan Li, Mingming Zhang

**Affiliations:** ^1^Department of Cardiology, Tangdu Hospital, The Fourth Military Medical University, Xi'an, China; ^2^Department of Orthodontics, School of Stomatology, The Fourth Military Medical University, Xi'an, China

## Abstract

**Background:**

miR-205 is important for oxidative stress, mitochondrial dysfunction, and apoptosis. The roles of miR-205 in cardiac ischemia/reperfusion (I/R) injury remain unknown. The aim of this research is to reveal whether miR-205 could regulate cardiac I/R injury by focusing upon the oxidative stress, mitochondrial function, and apoptosis.

**Methods:**

Levels of miR-205 and Rnd3 were examined in the hearts with I/R injury. Myocardial infarct size, cardiac function, oxidative stress, mitochondria function, and cardiomyocyte apoptosis were detected in mice with myocardial ischemia/reperfusion (MI/R) injury. The primary neonatal cardiomyocytes underwent hypoxia/reoxygenation (H/R) to simulate MI/R injury.

**Results:**

miR-205 levels were significantly elevated in cardiac tissues from I/R in comparison with those from Sham. In comparison with controls, levels of Rnd3 were significantly decreased in the hearts from mice with MI/R injury. Furthermore, inhibiting miR-205 alleviated MI/R-induced apoptosis, reduced infarct size, prevented oxidative stress increase and mitochondrial fragmentation, and improved mitochondrial functional capacity and cardiac function. Consistently, overexpression of miR-205 increased infarct size and promoted apoptosis, oxidative stress, and mitochondrial dysfunction in mice with MI/R injury. In cultured mouse neonatal cardiomyocytes, downregulation of miR-205 reduced oxidative stress in H/R-treated cardiomyocytes. Finally, inhibiting Rnd3 ablated the cardioprotective effects of miR-205 inhibitor in MI/R injury.

**Conclusions:**

We conclude that inhibiting miR-205 reduces infarct size, improves cardiac function, and suppresses oxidative stress, mitochondrial dysfunction, and apoptosis by promoting Rnd3 in MI/R injury. miR-205 inhibitor-induced Rnd3 activation is a valid target to treat MI/R injury.

## 1. Introduction

Despite remarkable progress in disease prevention, diagnosis, and better control of risk factors, heart disease remains the most major contributor to mortality and morbidity worldwide [1, 2]. The myocardium that suffered from acute myocardial infarction (AMI) becomes ischemic and is consequently replaced by fibrosis [3]. Although ischemic myocardium can be treated by drugs or surgery, reperfusion causes damage to the heart, known as reperfusion injury [4]. Though coronary heart disease (CHD) mortality declined about 1%-1.8% annually in the past 20 years, thanks to percutaneous coronary intervention, estimated years of life lost because of MI are still very high [5]. Such circumstance indicates that great effort is still needed in research into alleviating cardiac I/R injury.

MicroRNAs (miRs) are small noncoding RNAs with sizes around 18–24 nucleotides, which could promote the translation or degradation of mRNAs and then regulate gene expression [6]. Based on this character, miRs involve in various biological process such as development, cancer, metabolic diseases, inflammation, and cardiovascular diseases [7–10]. Moreover, miRs also have the potential to be novel biomarkers and therapeutic agents [11, 12]. Specific to cardiac I/R injury, miRs often play its role through regulating genes expression in key signaling pathways. For example, miR-19a suppresses myocardial apoptosis in I/R injury [13]. Besides, miR-20b-5p could downregulate Smad7, activate TGF-*β*/Smad pathway, and thus accelerate ventricular remodeling in I/R injury [14]. Therefore, our research decides to put emphasis on the miRs. miR-205-5p is one of the highly conserved miRNAs, located in the region of chromosome 1q32.2 of human genome. However, the role of miR-205-5p in MI/R injury still remains unclear.

Mitochondria suffer a deficiency to supply the cardiomyocyte with energy in MI/R injury [15]. Mitochondria dysfunction induced by MI/R injury leads to systolic dysfunction of heart because of insufficient energy [16]. The primary function of mitochondria is to produce abundant ATP, which is of critical importance for the normal work of heart. While in diseases, abnormalities of mitochondria are often discovered in form of mitochondrial enlargement, matrix derangement, and cristae loss, which all indicates abnormality of mitochondrial quality control [17]. Besides, oxidative stress is also an indicator of mitochondrial abnormality. Mitochondrial quality control includes mitochondrial biogenesis, mitochondrial dynamics, and mitophagy [18]. Kubli et al. generated parkin-deficient mice, which displayed impaired mitophagy and were more sensitive to myocardial infarction [19]. Mitochondrial quality surveillance may be a therapeutic target in myocardial infraction [20]. Taken together, this evidence demonstrate that cardiac restoration post injury is greatly related to mitochondrial function.

Oxidative stress originated from the overwhelmed ROS and the insufficient antioxidant defense systems [21]. The generation of ROS is promoted during I/R injury [22]. Oxidative stress resulted in alterations in protein function and oxidation of mitochondrial DNA. At the same time, mitochondrial damage induced the generation of ROS [23]. Ultimately, oxidative stress activates caspases and promotes cell apoptosis [24].

Small guanosine triphosphatases (GTPases) are enzymes which can hydrolyze guanosine triphosphate (GTP), and the most well-known GTPase family is Ras GTPases [25]. RND3, also called as RhoE, belongs to Ras homologous (Rho) family 31. Different from other GTPases, RND3 is lack of GTPase activity, but it is actively involved in actin cytoskeletal dynamics, apoptosis, differentiation, and other physiological process [26, 27]. Based on the discovered downregulation of RND3 in human falling heart, Yue et al. generated Rnd3^+/−^ haploinsufficient mice. Compared to wild-type ones, Rnd3^+/−^ mice displayed apoptotic cardiomyopathy when exposed to pressure overload. Researchers also found that total deletion of RND3 would result in embryonic death because of fetal arrhythmias [28, 29]. While mice with RND3 knockdown could survive to adulthood, knockdown of RND3 would disturb the ubiquitination of *β*2-adrenergic receptor and finally lead to irregular spontaneous Ca^2+^ release [30]. In addition to above, under stress, RND3 could stabilize HIF1*α* and promote VEGF expression [31]. This cardioprotective effect of RND3 is evidenced by reserved cardiac function of Rnd3 transgenic mice exposed to pressure overload. All these data illustrate that RND3 exert multiple effects in various cardiovascular diseases. Based on TargetScan, the RND3 is the target of miR-205-5p. However, no efforts have been made to explore the effects of miR-205/Rnd3 in MI/R injury.

## 2. Research Design and Methods

### 2.1. Ethics and Subjects

The experiments were performed in adherence with the National Institutes of Health Guidelines on the Use of Laboratory Animals and were approved by the Fourth Military Medical University Ethic Committee on Animal Care.

### 2.2. Experiment Protocols

Male C57BL/6 mice of 6-8 weeks were randomly divided into the following groups with *n* = 6 each: (1) Sham; (2) MI/R+NC; (3) MI/R+miR-205 inhibitor; (4) MI/R+Control mimic; and (5) MI/R+miR-205 mimic. To illustrate whether miR-205 regulates cardiac I/R injury through Rnd3, mice were randomized to receive one of the following treatments with *n* = 6 each: (1) MI/R; (2) MI/R+AAV9-sh-Rnd3; (3) MI/R+miR-205 inhibitor; and (4) MI/R+miR-205 inhibitor+AAV9-sh-Rnd3. MI/R model construction was performed as previously described [32]. A 6–0 silk suture slipknot was placed at the proximal one-third of the left anterior descending artery. After 30 minutes of ischemia, the slipknot was released, and the myocardium was reperfused for 3 h.

### 2.3. Intracardiac Injection of miRNA Mimics and AAV9-sh-Rnd3

Mice were randomly subjected to intracardiac injection of miR-205 mimics or inhibitors (10 *μ*g per mouse heart), respectively, before MI/R. miR-205 mimics or inhibitors (10 *μ*g per heart) in a total volume of 50 *μ*l were injected immediately before the ligation of LAD coronary artery. The miRNA mimics, miRNA inhibitors, and AAV9-sh-Rnd3 (2 × 10^11^ viral genome particles per mouse heart) were evenly injected into three sites around the infarcted area (anterior wall, lateral wall, and apex area).

### 2.4. Primary Cardiomyocytes Culture and In Vitro Simulated Ischemia/Reperfusion Model Construction

Primary cardiomyocytes were isolated and cultured as previously described. The hearts of 1- to 3-day-old mice were isolated. The ventricular tissue was cut into pieces and digested with 5 ml collagenase type II at a concentration of 1 mg/ml for 7 min. The supernatant was fed into a 15 ml centrifuge tube, and the digestion was terminated with the same amount of DMEM and 10% fetal bovine serum (FBS). The above steps were repeated until the heart tissue is completely digested. The isolated cells were cultured in a 37°C culture flask for 2 hours to enrich the culture with cardiomyocytes. The nonadherent cardiomyocytes were collected and then plated onto gelatin-coated plated. For induction of simulated I/R injury, cells were cultured in D-Hanks solution in a modular incubator chamber (Biospherix) with 1% O_2_, 5% CO_2_, and 94% N_2_ for 4 h (simulated ischemia for 4 h), then exposed to atmosphere of 21% O_2_, 5% CO_2_, and 74% N_2_, and cultured for 4 h (simulate reperfusion for 4 h). Primary cardiomyocytes were randomly divided into the following groups: (1) Con; (2) H/R+NC; (3) H/R+miR-205 inhibitor; (4) H/R+Control mimic; and (5) H/R+miR-205 mimic. To illustrate whether miR-205 regulates H/R injury in cardiomyocytes through Rnd3, cardiomyocytes were randomized to receive one of the following treatments: (1) H/R; (2) H/R+Ad-sh-Rnd3; (3) H/R+miR-205 inhibitor; and (4) H/R+miR-205 inhibitor+Ad-sh-Rnd3.

### 2.5. Measurement of Myocardial Infarct Size

Myocardial Infarct Size was evaluated by Evans Blue/TTC staining as previously described [32].

### 2.6. Determination of Myocardial Apoptosis

Myocardial apoptosis was determined by terminal deoxynucleotidyl transferase-mediated dUTP-biotin nick end labeling (TUNEL) staining as previously described [32].

### 2.7. Determination of Cardiac Function

Echocardiography was performed at 24 h after reperfusion as previously described [32].

### 2.8. Calcium Retention Capacity (mCRC)

The mitochondrial calcium retention capacity (mCRC) was detected as previously described [29].

### 2.9. ROS Production and MnSOD Activity

The production of ROS was measured as previously described [33]. MnSOD was assayed as previously described [33].

### 2.10. Western Blot Evaluation

Total proteins from cardiomyocytes were separated by SDS-PAGE, blotted and probed with anti-*β*-actin antibody (Santa Cruz, CA, USA), anti-Rnd3 (Cell Signaling, Danvers, MA, USA), anti-Cleaved Caspase-3, and anti-Cleaved Caspase-9 (Sigma, St. Louis, MO, USA). The signals were quantified by densitometry and normalized to *β*-actin.

### 2.11. Citrate Synthase (CS), ATP Content, Mitochondria Isolation, Immunostaining Assay, and Transmission Electron Microscopy (TEM)

Citrate synthase and the ATP content of the myocardium were measured as previously described [29]. Mitochondria isolation, immunostaining assay, and transmission electron microscopy (TEM) were performed as previously described [30].

### 2.12. Mitochondrial Membrane Potential (ΔΨ) Detection

The ΔΨ of cardiomyocytes was assessed by the JC-1 assay kit (Beyotime, CHINA) [34].

### 2.13. Statistical Analysis

Continuous variables that approximated the normal distribution were expressed as means ± SD. Comparison between groups was subjected to ANOVA followed by the Bonferroni correction for post hoc *t*-test. Data expressed as proportions were assessed with a Chi-square test. Two-sided tests have been used throughout, and *p* values < 0.05 were considered statistically significant. The SP4SS software package version 17.0 (SPSS, Chicago, IL) was used for data analysis.

## 3. Results

### 3.1. miR-205 Inhibitor Alleviates, while miR-205 Mimic Administration Aggravates Cardiac MI/R Injury in Mice

**Figure 1:**
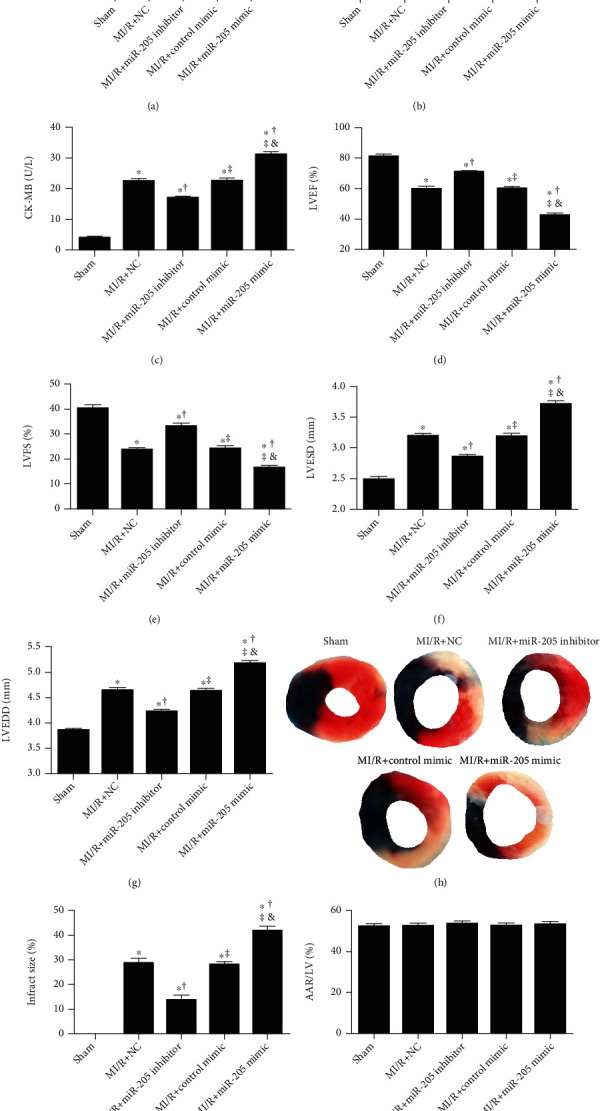
miR-205 inhibitor alleviates, while miR-205 mimic administration aggravates cardiac MI/R injury in mice. (a) Relative expression of miRNA-205. (b, c) Lactate dehydrogenase (LDH) and creatine kinase-MB (CK-MB) release after myocardial I/R injury in mice. (d–g) Left ventricular ejection fraction (LVEF), left ventricular fraction shortening (LVFS), left ventricular end systolic diameter (LVESD), and left ventricular end diastolic diameter (LVEDD) measured by echocardiography. (h) Representative images of infarct size as stained by Evans Blue and TTC. (i, j) Quantitative analysis of infarct size and AAR/LV at 3 h after I/R injury in mice. *n* = 6 in each group. The columns and errors bars represent means and SD. ^∗^*p* < 0.05 vs. Sham, ^†^*p* < 0.05 vs. MI/R+NC, ^‡^*p* < 0.05 vs. MI/R+miR-205 inhibitor, ^&^*p* < 0.05 vs. MI/R+Control mimic.

miR-205 was significantly increased in the MI/R group (Figure 1(a)). Compared with the MI/R group, LDH and CK-MB were significantly decreased in the miRNA-205 inhibitor group, while increased in the miRNA-205 mimic group (Figures 1(b) and 1(c)). Echocardiography showed that the significant increase of cardiac function markers LVEF and LVFS and the significant decrease of LVESD and LVEDD were observed in the MI/R+miR-205 inhibitor group (Figures 1(d)–1(g)). Furthermore, miR-205 mimic significantly decreased LVEF and LVFS and increased LVESD and LVEDD compared with the MI/R group (Figures 1(d)–1(g)). Representative images of infarct size are shown in Figure 1(h). miR-205 inhibitor significantly decreased infarct size after MI/R injury compared with the MI/R group (Figure 1(i)). Meanwhile, miR-205 mimic administration significantly increased infarct size after MI/R injury compared with the MI/R group (Figure 1(i)).  Figure 1(j) reveals that there was no significant difference in the ratio of area at risk (AAR) to left ventricle (LV) area among groups.

### 3.2. miR-205 Inhibitor Improves, while miR-205 Mimic Administration Aggravates Mitochondrial Dysfunction and Oxidative Stress in Mice that Underwent MI/R Injury

**Figure 2:**
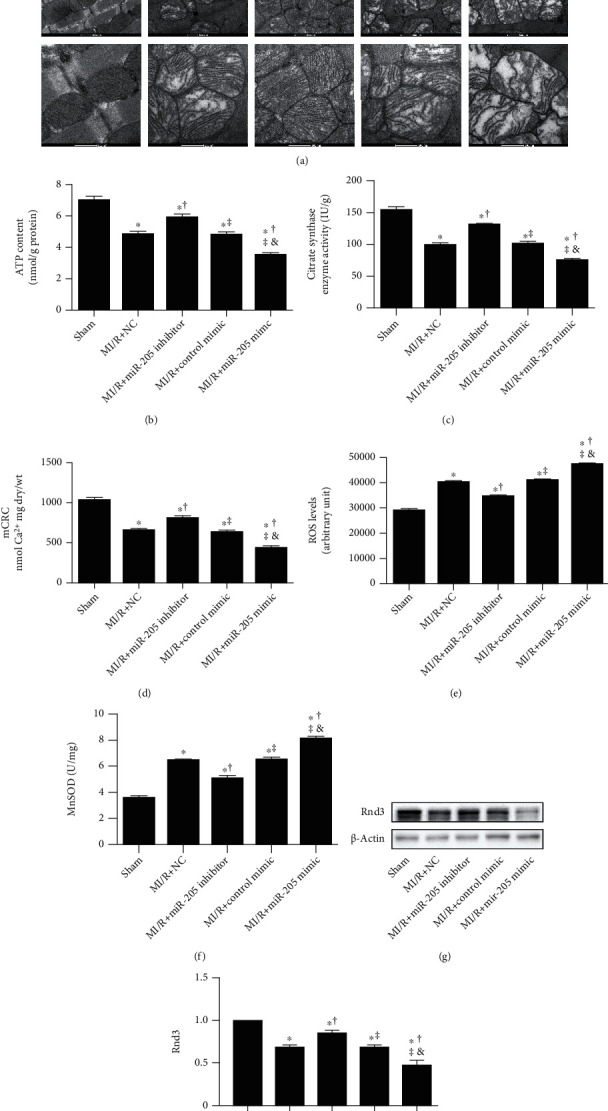
miR-205 inhibitor improves, while miR-205 mimic administration aggravates mitochondrial dysfunction and oxidative stress in mice that underwent MI/R injury. (a) Mitochondria morphological defects (magnification: upper panel ×9900; middle panel ×20500; lower panel ×43000). (b, c) ATP content and citrate synthase (CS) activity in the ischemic myocardium in mice subjected to MI/R injury. (d) Sensitivity of the mitochondrial permeability transition pore (mPTP) opening to calcium as evidenced by mCRC measurement. (e) ROS levels assessed by EPR spectroscopy. (f) Mitochondrial MnSOD activity. (g, h) Western blot analysis of Rnd3 expression. *n* = 6 in each group. ^∗^*p* < 0.05 vs. Sham, ^†^*p* < 0.05 vs. MI/R+NC, ^‡^*p* < 0.05 vs. MI/R+miR-205 inhibitor, ^&^*p* < 0.05 vs. MI/R+Control mimic.

TEM revealed that miRNA-205 inhibitor treatment alleviated mitochondrial structural damage after MI/R injury (Figure 2(a)). Compared to the MI/R group, mitochondrial ATP content (Figure 2(b)) and CS activity (Figure 2(c)) were significantly elevated in the MI/R+miRNA-205 inhibitor group and decreased in the MI/R+miRNA-205 mimic group. Compared to the MI/R group, the mCRC in the MI/R+miRNA-205 inhibitor group was significantly enhanced (Figure 2(d)). ROS levels (Figure 2(e)) and mitochondrial MnSOD activity (Figure 2(f)) were significantly decreased in the miR-205 inhibitor-injected hearts, while increased in the miRNA-205 mimic-injected hearts. Moreover, miRNA-205 inhibitor increased, while miRNA-205 overexpression administration decreased the expression of Rnd3 (Figures 2(g) and 2(h)).

### 3.3. Inhibiting miR-205 Improves, while miR-205 Overexpression Administration Aggravates Apoptosis in Mice that Underwent Cardiac MI/R Injury

**Figure 3:**
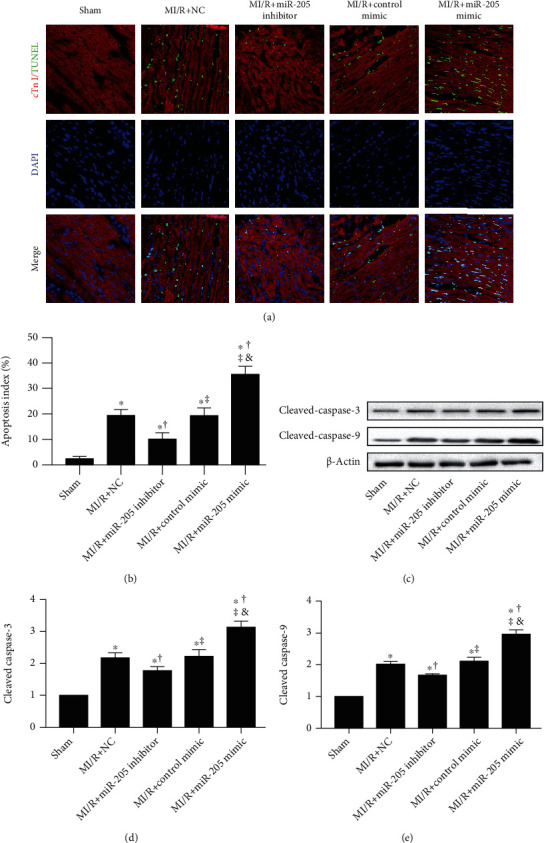
Inhibiting miR-205 improves, while miR-205 overexpression administration aggravates apoptosis in mice that underwent cardiac MI/R injury. (a, b) Representative images of TUNEL staining and percentage of TUNEL-positive nuclei, scale bars = 50 *μ*m. (c–e) Western blot analysis of cleaved caspase-3 and cleaved caspase-9 expression. *n* = 6 in each group. ^∗^*p* < 0.05 vs. Sham, ^†^*p* < 0.05 vs. MI/R+NC, ^‡^*p* < 0.05 vs. MI/R+miR-205 inhibitor, ^&^*p* < 0.05 vs. MI/R+Control mimic.

TUNEL-positive cardiomyocytes in the MI/R+miRNA-205 inhibitor group were less frequently observed compared with the MI/R group (Figures 3(a) and 3(b)). miRNA-205 mimic increased the apoptosis rate after MI/R injury (Figures 3(a) and 3(b)). Concomitantly, miRNA-205 inhibitor administration decreased cleaved caspase-3 and cleaved caspase-9 after MI/R injury (Figures 3(c)–3(e)). miRNA-205 mimic administration increased cleaved caspase-3 and cleaved caspase-9 after MI/R injury (Figures 3(c)–3(e)).

### 3.4. Inhibiting RND3 Ablated the Cardioprotective Effects of miRNA-205 Inhibitor

**Figure 4:**
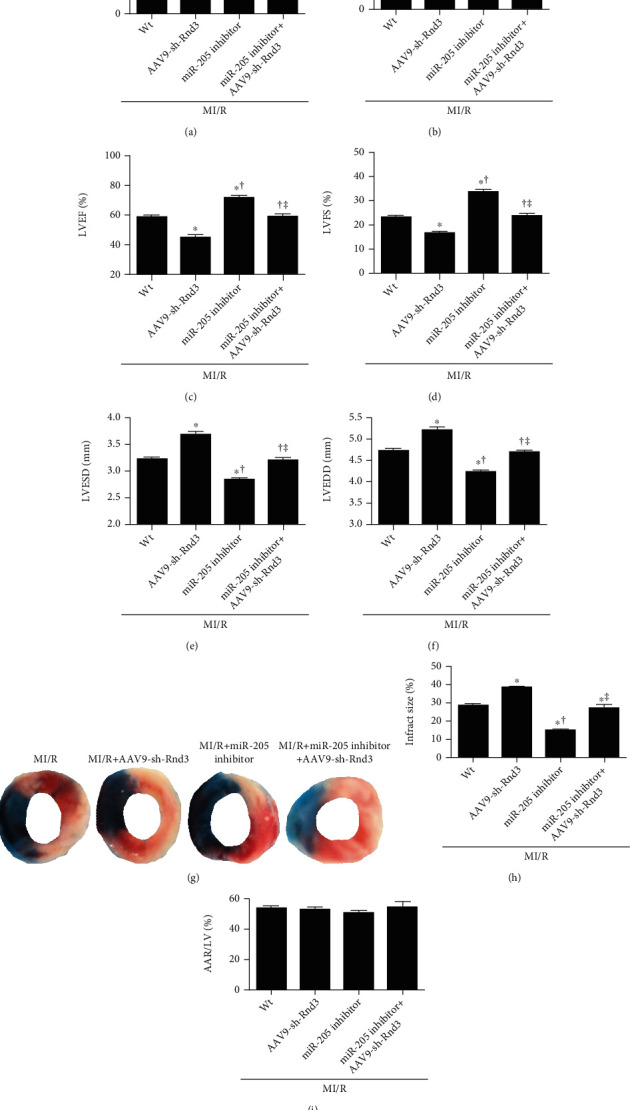
Inhibiting RND3 ablated the cardioprotective effects of miRNA-205 inhibitor. (a, b) Lactate dehydrogenase (LDH) and creatine kinase-MB (CK-MB) release. (c–f) Left ventricular ejection fraction (LVEF), left ventricular fraction shortening (LVFS), left ventricular end systolic diameter (LVESD), and left ventricular end diastolic diameter (LVEDD). (g) Representative images of infarct size as stained by Evans Blue and TTC. (h, i) Infarct size. AAR/LV had no statistical difference between groups 3 h after I/R injury. *n* = 6 in each group. The columns and errors bars represent means and SD. ^∗^*p* < 0.05 vs. MI/R, ^†^*p* < 0.05 vs. MI/R+AAV9-sh-Rnd3, ^‡^*p* < 0.05 vs. MI/R+miR-205 inhibitor.

**Figure 5:**
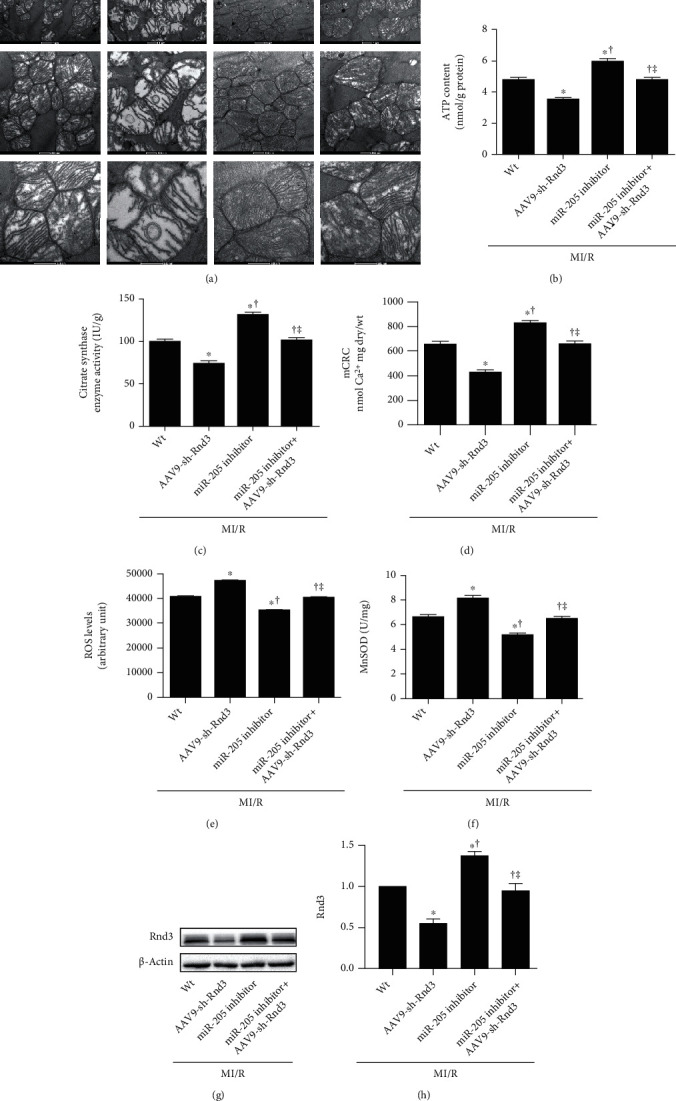
Inhibiting RND3 ablated the cardioprotective effects of miRNA-205 inhibitor in mitochondrial dysfunction and oxidative stress in mice that underwent MI/R injury. (a) Mitochondria morphological defects (magnification: upper panel ×9900; middle panel ×20500; lower panel ×43000). (b, c) ATP content and citrate synthase (CS) activity in the ischemic myocardium in the isolated mitochondrial in mice subjected to cardiac I/R injury. (d) Sensitivity of the mitochondrial permeability transition pore (mPTP) opening to calcium as evidenced by mCRC measurement. (e) ROS levels assessed by EPR spectroscopy. (f) Mitochondrial MnSOD activity. (g, h) Western blot analysis of Rnd3 expression. *n* = 6 in each group. ^∗^*p* < 0.05 vs. MI/R, ^†^*p* < 0.05 vs. MI/R+AAV9-sh-Rnd3, ^‡^*p* < 0.05 vs. MI/R+miR-205 inhibitor.

**Figure 6:**
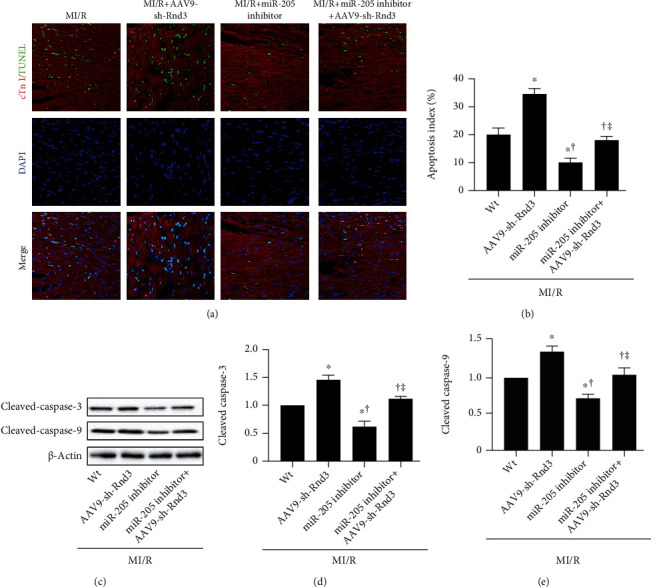
Inhibiting RND3 ablated the cardioprotective effects of miRNA-205 inhibitor in apoptosis in mice that underwent cardiac MI/R injury (a, b) Representative images of TUNEL staining and percentage of TUNEL-positive nuclei, scale bars = 50 *μ*m. (c–e) Western blot analysis of cleaved caspase-3 and cleaved caspase-9 expression. *n* = 6 in each group. ^∗^*p* < 0.05 vs. MI/R, ^†^*p* < 0.05 vs. MI/R+AAV9-sh-Rnd3, ^‡^*p* < 0.05 vs. MI/R+miR-205 inhibitor.

To elucidate the mechanism of miR-205 on MI/R injury in mice, we checked the TargetScan and find Rnd3. AAV9-sh-Rnd3 was injected to the mice with MI/R injury. As is shown in Figures 4(a) and 4(b), a significant increase of cardiac injury markers LDH and CK-MB was observed in AAV9-sh-Rnd3-injected mice after MI/R injury. The infarct size was significantly larger in the AAV9-sh-Rnd3-injected mice compared with the MI/R mice (Figures 4(g)–4(i)). Additionally, we observed injured cardiac function, as indicated by decreased LVEF and LVFS and increased LVESD and LVEDD in MI/R+AAV9-sh-Rnd3 mice (Figures 4(c)–4(f)). Interestingly, miR-205 inhibitor did not exhibit protective effects in AAV9-sh-Rnd3-injected mice with MI/R injury, as evidenced by infract size and cardiac function (Figure 4). The result of mitochondrial function, oxidative stress, and apoptosis was consistent with the above results (Figures 5 and 6). miR-205 inhibitor alleviated the mitochondria ultrastructure disorder in MI/R hearts but not in MI/R+miR-205 inhibitor+AAV9-sh-Rnd3 hearts (Figure 5(a)). AAV9-sh-Rnd3 significantly further decreased ATP content and CS activity in the MI/R+AAV9-sh-Rnd3 group, while miR-205 inhibitor insignificantly increased ATP content and CS activity in the MI/R+miR-205 inhibitor+AAV9-sh-Rnd3 group (Figures 5(b) and 5(c)). The result of mCRC, ROS levels, and mitochondrial MnSOD activity was consistent with above results. Consistently, AAV9-sh-Rnd3 treatment decreased mCRC and increased ROS levels, and mitochondrial MnSOD activity underwent MI/R injury. AAV9-sh-Rnd3 treatment decreased the expression of Rnd3 in the MI/R+miR-205 inhibitor+AAV9-sh-Rnd3 hearts (Figures 5(g) and 5(h)). Coincidentally, AAV9-sh-Rnd3 injection increased the TUNEL-positive cardiomyocytes and the expression of cleaved caspase-3 and cleaved caspase-9 (Figures 6(a)–6(e)). However, the miR-205 inhibitor did not decrease cardiomyocyte apoptosis in the presence of AAV9-sh-Rnd3 after MI/R injury. The miR-205 inhibitor had no effect on the cleaved caspase-3 and cleaved caspase-9 in the MI/R mice subjected to AAV9-sh-Rnd3 injection (Figures 6(a)–6(e)). All these results indicate that miR-205 inhibitor alleviates MI/R injury by promoting Rnd3 expression.

### 3.5. Inhibiting miR-205 Improves H/R-Induced Oxidative Stress, while Inhibiting Rnd3 Ablated the Cardioprotective Effects of miR-205 Inhibitor in Primary Cardiomyocytes

**Figure 7:**
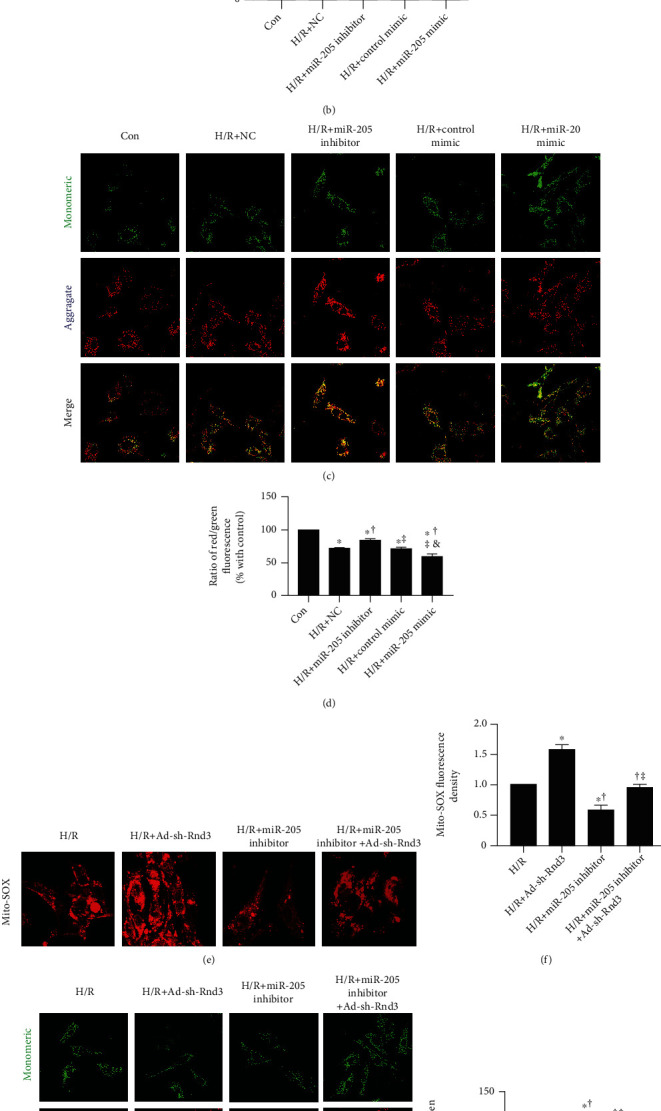
Inhibiting miR-205 improves H/R-induced oxidative stress, while inhibiting RND3 ablated the cardioprotective effects of miR-205 inhibitor in primary cardiomyocytes. (a, b) Representative images of mitochondrial ROS in primary cardiomyocytes, scale bars = 50 *μ*m. (c, d) Representative images of JC-1 and the ratio of aggregated (red) and monomeric (green) in neonatal mice cardiomyocytes, scale bars = 20 *μ*m. ^∗^*p* < 0.05 vs. Con, ^†^*p* < 0.05 vs. H/R+NC, ^‡^*p* < 0.05 vs. H/R+miR-205 inhibitor, ^&^*p* < 0.05 vs. H/R+Control mimic. (e, f) Representative images of mitochondrial ROS in neonatal mice cardiomyocytes, scale bars = 50 *μ*m. (g, h) Representative images of JC-1 and the ratio of aggregated (red) and monomeric (green) in neonatal mice cardiomyocytes, scale bars = 20 *μ*m. The number of cardiomyocytes was counted (*n* = 50 in each group). ^∗^*p* < 0.05 vs. H/R, ^†^*p* < 0.05 vs. H/R+AAV9-sh-Rnd3, ^‡^*p* < 0.05 vs. H/R+miR-205 inhibitor.

Consistent with these observations, miR-205 inhibitor significantly decreased, while miR-205 mimic increased the level of mitochondrial ROS, which was measured by a MitoSOX kit, in cardiomyocytes compared with the H/R group (Figures 7(a) and 7(b)). JC-1 fluorescence images revealed that miR-205 inhibitor increased the ΔΨ, while miR-205 mimic reduced the ΔΨ in cardiomyocytes that underwent H/R injury (Figures 7(c) and 7(d)). Moreover, Ad-sh-Rnd3 increased the level of mitochondrial ROS in cardiomyocytes that underwent H/R injury. However, miR-205 inhibitor reduced oxidative stress in cardiomyocyte after H/R, which almost disappeared after downregulation of Rnd3 (Figures 7(e) and 7(f)). JC-1 fluorescence images reveal that Ad-sh-Rnd3 reduced the ΔΨ in cardiomyocytes that underwent H/R injury, and it could block the effect of miR-205 inhibitor on the increase of ΔΨ that underwent H/R injury (Figures 7(g) and 7(h)).

## 4. Discussion

The morbidity and mortality of patients with AMI are high worldwide [35]. In patients with MI, the myocardial reperfusion treatment for salvaging viable myocardium, limiting MI size, and decreasing mortality is timely and effective [36]. However, reperfusion itself induces cardiac injury, named as reperfusion injury, and there is still lack of effective therapy for MI/R injury. It has always been a great threat to human's health and life. Numerous studies and clinical evidence support the notion that miRNAs play essential roles in cardiovascular diseases, such as myocardial infarction, cardiac hypertrophy, cardiomyopathy, and arrhythmias, and could be used for the diagnosis and prevention of cardiovascular diseases. miR-205 is discovered to be a suppressor factor in breast cancer, which can target E2F transcription factor 1, angiomotin, and other genes, and then to reduce cell proliferation, inhibit invasion, and increase apoptosis. Ling et al. found that miR-205 was markedly inhibited in air pollution that induced myocardial inflammation, and the inhibition of miR-205 activated the IRAK2/TRAF6/NF-*κ*B signaling pathway [37]. Except these, miR-205 is also found to be increased in animals treated with imatinib mesylate and doxorubicin and animals with chronic heart failure [38, 39]. We found that inhibiting miR-205 improved cardiac dysfunction and mitochondrial dysfunction and reduced infarct size, oxidative stress, and apoptosis by promoting Rnd3 in MI/R injury. The results suggested that miR-205 is detrimental in MI/R injury. Moreover, the cardiac protective effects of miR-205 inhibitor are abolished through inhibiting Rnd3.

Mitochondrial dysfunction leads to contractile dysfunction and pathological ventricular remodeling, which is associated with heart failure and mortality of patients. In the current study, we found that miR-205 inhibitor improved mitochondrial dysfunction, which was associated with impaired cardiac function. Meanwhile, miR-205 mimic exaggerated contractile dysfunction and mitochondrial dysfunction. The alterations in the mitochondrial function suggested that miR-205 inhibitor may play a protective role in pathological process of MI/R injury. Moreover, these protective effects of miR-205 inhibitor were abolished by knocking down Rnd3, which was in line with our previous study.

Oxidative stress has been identified as a major cause of cardiac injury in cardiovascular system. Under pathological conditions, excessive production of ROS impairs the balance between ROS and antioxidant substance, which is called oxidative stress. Oxidative stress results in negative effects on normal cardiac structure and cardiometabolic homeostasis [40]. Increased oxidative stress is implicated in MI/R injury, contractile dysfunction, mitochondrial dysfunction, myocyte apoptosis, and the progressive downward spiral of heart failure [41–44]. Cytoplasm, mitochondria, and peroxisomes are main sources of ROS. NADPH oxidase (NOX) family and mitochondrial complexes I-III are the most well-known contributors of production of cytoplasmic ROS and mitochondrial ROS, respectively. Overall, detrimental effects of excessive ROS in heart are attributed to dysfunction in electrophysiology, contractibility, energy metabolism, and fibrosis [45]. Excessive ROS could directly modify proteins involved in potassium channels, sodium-calcium exchanger, and other important ion channel and thus influence electrophysiology in heart [46]. Oxidative stress was increased in cardiac hypertrophy, and ROS-mediated activation of MAPKs and NF-*κ*B was discovered [47]. Our study has demonstrated that the miR-205 mimic exacerbates ROS level and subsequent increased superoxide generation. Furthermore, the current study demonstrated a substantial reduction of MI/R-induced ROS after miR-205 inhibitor treatment. Oxidative stress was significantly upregulated in MI/R mice with Rnd3 knockdown. Taken together, these results indicate that miR-205 inhibitor inhibited, while miR-205 mimic promoted the oxidative stress during exposure to MI/R injury.

The cardiomyocyte apoptosis is the chief player in MI/R injury [48]. Previous studies have revealed that downregulating miR-205 reduces myocardial apoptosis in rats with chronic heart failure [39]. In the current study, miR-205 inhibitor inhibited, while miR-205 mimic promoted cardiomyocyte apoptosis.

Rnd3, a member of the Rnd family, has been proved as a key factor in the pathophysiology process of cardiomyopathy, heart failure, and cancer [27]. Rnd3 can reduce microvascular leakage after injury [49]. Recent research suggested that coronary microvascular may be a new frontier in cardioprotection after MI/R injury [50]. Further evidence has revealed that insufficient Rnd3 results in apoptotic cardiomyopathy with heart failure. In cardiac I/R injury, RND3 deficiency promotes some proinflammatory gene expressions including tumor necrosis factor (TNF) superfamily and interferons. While cardiac RND3 overexpression inhibits inflammation post-MI and improves cardiac function [51]. Increased evidence suggested an essential role for myocardial Rnd3 in modulating cardiac function. Although the precise roles remain uncertain, previous studies have revealed that Rnd3 also mediates obesity and insulin resistance [52]. miR-205 was significantly increased in mice with I/R injury, whereas the expression of Rnd3 was decreased. Meanwhile, Rnd3 knockdown abolished the cardioprotective effect in MI/R injury after miR-205 inhibitor treatment, suggesting that miR-205 inhibitor alleviates the MI/R injury by promoting Rnd3. Furthermore, our study demonstrated that Rnd3 knockdown exhibited exacerbated cardiac systolic dysfunction and mitochondrial dysfunction and increased oxidative stress and apoptosis. We observed that miR-205 inhibitor decreased the infarct size, oxidative stress, and cardiomyocyte apoptosis and improved the mitochondrial function, which was abolished by downregulating Rnd3. Taken together, these results support Rnd3 as the primary downstream of miR-205 which maintains cardiac function in the MI/R.

In conclusion, we provided evidence that miR-205 inhibitor alleviated the cardiac I/R injury. In addition, these cardiac protective effects of miR-205 inhibitor are largely attributable to the Rnd3 activation. Although these data collectively indicate that miR-205 Inhibitor as a therapeutic target for MI/R injury, further studies are needed to test the clinical implications of miR-205 inhibitor in protecting against cardiac I/R injury.

## Data Availability

The data used to support the findings of this study are available from the corresponding authors upon request.
